# Transforming the tradition of discrete milk yield correction factors: A continuous 1-step DeLorenzo-Wiggans method

**DOI:** 10.3168/jdsc.2024-0583

**Published:** 2024-08-16

**Authors:** Xiao-Lin Wu, Malia J. Caputo, George R. Wiggans, H. Duane Norman, Asha M. Miles, Curtis P. Van Tassell, Ransom L. Baldwin, Michael M. Schutz, Javier Burchard, João Dürr

**Affiliations:** 1 Council on Dairy Cattle Breeding, Bowie, MD 20716; 2 Department of Animal and Dairy Sciences, University of Wisconsin–Madison, Madison, WI 53706; 3 USDA Animal Genomics and Improvement Laboratory, Beltsville, MD 20705; 4 Department of Animal Science, University of Minnesota, St. Paul, MN 55108

## Abstract

Over the past decades, various methods have been proposed to estimate daily milk yields from partial yields. Many of these methods divide milking interval time into varied classes, assuming that the yield correction factors are constant within classes but vary between classes. The DeLorenzo and Wiggans (D-W) method has been widely used in the United States, typically following a 2-step process. It calculates discrete yield factors for segmented milking interval classes and then refines them through a follow-up smoothing step. Such a 2-step approach is computationally inefficient, and discrete yield correction factors introduce biases. This study explored strategies to integrate continuous yield factors into established methods, exemplified by the D-W method. The renovated method, also called the polynomial-interaction regression model, postulates multiplicative yield correction factors as a linear or quadratic function of milking interval time, operating on interactions with partial yields. It uses all available data in a single step, exhibiting greater computability efficiency and higher estimation accuracy. A reparameterization leads to a linear model, making estimating the model parameters convenient. We evaluated the performance of the revised methods using a previous dataset of milking records from Holstein cows compared with some existing methods. The results showed that the refurbished model gave more accurate estimates of daily milk yields.

## Highlights

• We propose a 1-step approach, eliminating the necessity for follow-up smoothing of MCF.
• MCF is a linear or quadratic function of milking interval time, operating on interactions with partial yields.
• The renovated method can use all available data, not limited to specific milking interval classes.
• A reparameterization leads to a linear model, making estimating the model parameters convenient.
• Accuracy is enhanced by 1% to 2% on average and more significant when evaluated individually.

## Graphical Abstract

**Figure:**
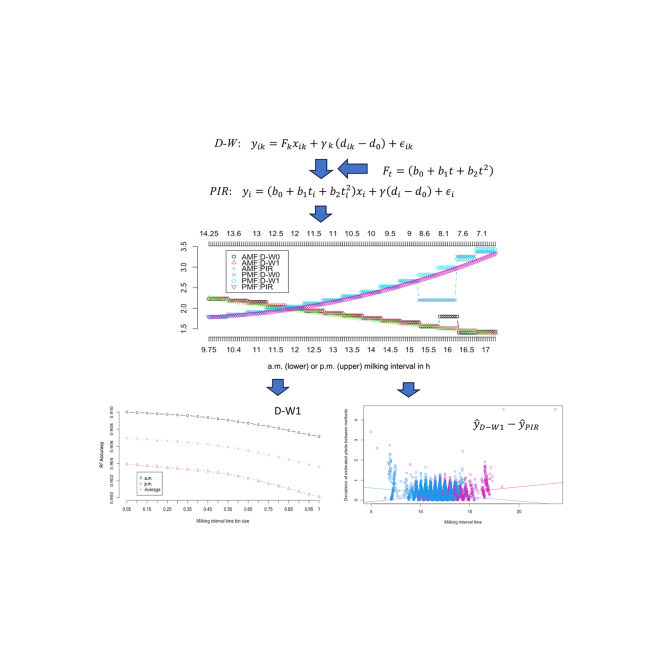
**Summary:** Over the past decades, various methods have been proposed to estimate daily milk yields from partial yields. Many of these methods divide milking interval time into varied classes, assuming that yield correction factors are constant within classes but vary between classes. A widely used method was proposed by DeLorenzo and Wiggans (1986), featuring a 2-step process. It calculates discrete yield factors for segmented milking interval classes and then refines them through a follow-up smoothing step. Such a 2-step approach is computationally inefficient, and using discrete multiplicative correction factors (MCF) introduces biases. This study explored strategies to integrate continuous yield factors into established methods, exemplified by the DeLorenzo- Wiggans (D-W) method. The renovated method can effectively use all available data in a single step, thus exhibiting greater computability efficiency and higher estimation accuracy.

The 1960s marked an important shift in milk testing plans in the United States and other countries. The transition moved from the standard supervised twice-daily, monthly testing scheme toward cost-efficient milk sampling plans, motivated to minimize costs associated with DHIA supervisor visits. One common approach is the a.m.-p.m. milk sampling method, which alternates milk sampling between morning (a.m.) or evening (p.m.) on test-days across lactation. Historically, each test-day milk yield was calculated as twice a single yield. Such a practice assumes equal milk secretion rates for a.m. and p.m. milkings and equal a.m. and p.m. milking sessions, each spanning precisely 12 h apart. In the case of unequal a.m. and p.m. milking intervals, the biases are assumed to be offset by complementary unevenness between a.m. and p.m. milkings. However, these assumptions are not practically plausible (Putnam and Gilmore, 1970).

Various statistical methods have been proposed for daily milk yield computations from partial daily yields (reviewed by Wu et al., 2023c). Traditionally, these methods assume that yield correction factors are constant within milking interval classes (**MICL**) but vary between classes. One widely used method in the United States was proposed by DeLorenzo and Wiggans (**D-W**; 1986) to derive multiplicative correction factors (**MCF**) for cows milked twice daily. First, it divides the entire milking interval time range into various classes of equal length and calculates MCF for each specific MICL. Then, these MCF are smoothed through a linear or quadratic polynomial function. In practice, such a 2-step approach is often computationally inefficient, and discrete MCF introduces estimation biases (reviewed by Wu et al., 2023c). In this study, we explored strategies to integrate continuous correction factors into existing methods, exemplified by the D-W method. The model renovation involved replacing separate local regressions with a single global linear or quadratic regression, operating on the interactions with partial yields.

The traditional D-W method fits separate linear regressions without an intercept, one at a time for each MICL, as follows:

[1] *y_ik_* = *F_k_x_ik_* + *ε_ik_*.

Here, *y_ik_* and *x_ik_* are the total and partial (a.m. or p.m.) daily yield, respectively, for a particular animal *i* measured on the *k*th discrete MICL, *F_k_* is the regression coefficient corresponding to the MCF specific to the *k*th MICL, and *ε_ik_* is the error term, assumed to have an expected value of 0, *E*(*ε_ik_*) = 0. Here, we omit the subscript for a.m. or p.m. milking as we initially present the method for analyzing a.m. and p.m. milkings separately. A joint analysis of a.m. and p.m. milkings is discussed later. The regression coefficient, *F_k_*, can also be expressed as the ratio of the expected value of a daily milk yield over the expected value of a partial yield.

Additional covariates and categorical variables can be included to account for secondary sources of variation in daily milk yields. For instance, incorporating DIM as a covariate leads to the following model:

[2] *y_ik_* = *F_k_x_ik_* + *γ_k_*(*d_ik_* − *d*_0_) + *ε_ik_*.

Here, *γ_k_* denotes the regression coefficient of DIM and *d*_0_ is a constant value, which can be the DIM mean or an empirical value (e.g., *d*_0_ = 158; DeLorenzo and Wiggans, 1986; Liu et al., 2000). Fitting separate linear regressions to various MICL with the D-W approach has disadvantages. First, MCF tend to fluctuate between neighboring MICL when directly obtained from separate linear regressions. Second, the data volume can vary substantially between MICL, and there may not be sufficient data to compute MCF reliably for all MICL, particularly for MICL featuring small and large milking interval times. Hence, after the initial model fitting, a smoothing step is often employed to reduce the fluctuation and impute MCF for MICL where the data information is insufficient. Smoothing is usually conducted by fitting the reciprocal of the “raw” MCF as a linear function (DeLorenzo and Wiggans, 1986) or as a quadratic polynomial function (Shook et al., 1980) of milking interval time (precisely speaking, midpoints of milking interval classes).

In this study, we proposed modifying the D-W method by introducing a 1-step procedure that leverages all available data information to calculate continuous MCF. This new approach employs a global regression that operates on the interactions between partial yields and milking interval time, contrasting the tradition of restricting MCF calculations to data specific to each MICL. First, we define continuous MCF as a function of milking interval time, *F_t_* = *f*(*t*), specific for every time point. Note that the switch of the subscript of *F* from *k* to *t* is fundamental, implying a transition from the traditional discrete MCF to continuous MCF with computational and modeling advantages. It allows the model to use all available data more effectively than the traditional methods that limit computing MCF to each specific MICL. Then, we applied a linear function for *F_t_* = *f*(*t*) in thrice-milkings, in which the milking interval time is mostly less than 12 h, and a quadratic function in twice-milkings, in which the milking interval time can go beyond 12 h.

Consider the quadratic form and include DIM as a covariate. Then, the D-W model is reformatted as the following:

[3] $$y_{i}=\left(b_{0}+b_{1}t_{i}+b_{2}t_{i}^{2}\right)x_{i}+\gamma \left(d_{i}-d_{0}\right)+\varepsilon_{i}.$$

Here, *t_i_* denotes the milking interval time for the *i*th animal. Including a quadratic term $\left(t_{i}^{2}\right)$ allows the model to account for the nonlinear effects of the time interval on the daily yield. Then, MCF are calculated pertaining to specific milking interval time (*t*) as follows:

[4] *F_t_* = *b*_0_ + *b*_1_*t* + *b*_2_*t*^2^.

Note that setting *b*_2_ = 0 yields a linear function for deriving MCF applicable to cows milked thrice daily or more frequently. Given a partial yield and computed continuous MCF, the corresponding daily is calculated as follows:

[5] $${\hat{y}}_{i}=F_{t}x_{i}+\hat{\gamma }\left(d_{i}-d_{0}\right),$$

where $F_{t}={\hat{b}}_{0}+{\hat{b}}_{1}t+{\hat{b}}_{2}t^{2}.$

In this paper, this modified D-W model is referred to as polynomial-interactions regression (**PIR**) because it operates on the interactions between partial milk yields and milking interval time in linear and quadratic forms. Mathematically, this new model is a nonlinear function, but all model parameters can conveniently be estimated with reparameterization. Let *τ_i_* = (*t_i_* × *x_i_*), which represents the interaction term between linear milking interval time and a partial yield pertaining to each animal. Similarly, let $\delta_{i}=t_{i}^{2}\times x_{i},$ for the interaction between quadratic milking interval time and a partial yield. Then, rearranging model [3] leads to the following:

[6] *y_i_* = *b*_0_*x_i_* + *b*_1_*τ_i_* + *b*_2_*δ_i_* + *γ*(*d_i_* – *d*_0_) + *ε_i_*.

Further model extensions are straightforward. For instance, a joint analysis of both milkings can be implemented as follows:

[7] $$y_{ij}=\left(b_{j0}+b_{j1}t_{ij}+b_{j2}t_{ij}^{2}\right)x_{ij}+\gamma \left(d_{ij}-d_{0}\right)+\varepsilon_{ij}.$$

Here, the subscript *j* indexes a.m. (*j* = 1) or p.m. (*j* = 2) milkings. The above model assumes heterogeneous linear and quadratic polynomial functions of milking interval time in interaction with an a.m. or p.m. partial yield and a common DIM effect for a.m. and p.m. milkings. However, various settings of the joint analysis model can be made possible depending on the data.

The performance of the renovated model was evaluated using a previous dataset (Wu et al., 2023b). This dataset consisted of 15,088 Holstein milking records (7,544 a.m. and 7,544 p.m.) collected from 3,717 animals. This dataset was randomly sampled from 23 herds in 11 states in the United States. The records covered the first 3 lactations (39.8%, 59.4%, and 0.8%) in 4 consecutive years (2006 to 2009). These 11 states represent 4 of the 5 geologic climate regions (https://codes.iccsafe.org/content/IECC2021P1/chapter-3-ce-general-requirements). The blending of milking records was also intended to average out the effects of secondary variables such as DIM, parities, and geological regions. The distributions of a.m. or p.m. milking records per 30-min milking time bin are shown in Figure 1.

**Figure 1:**
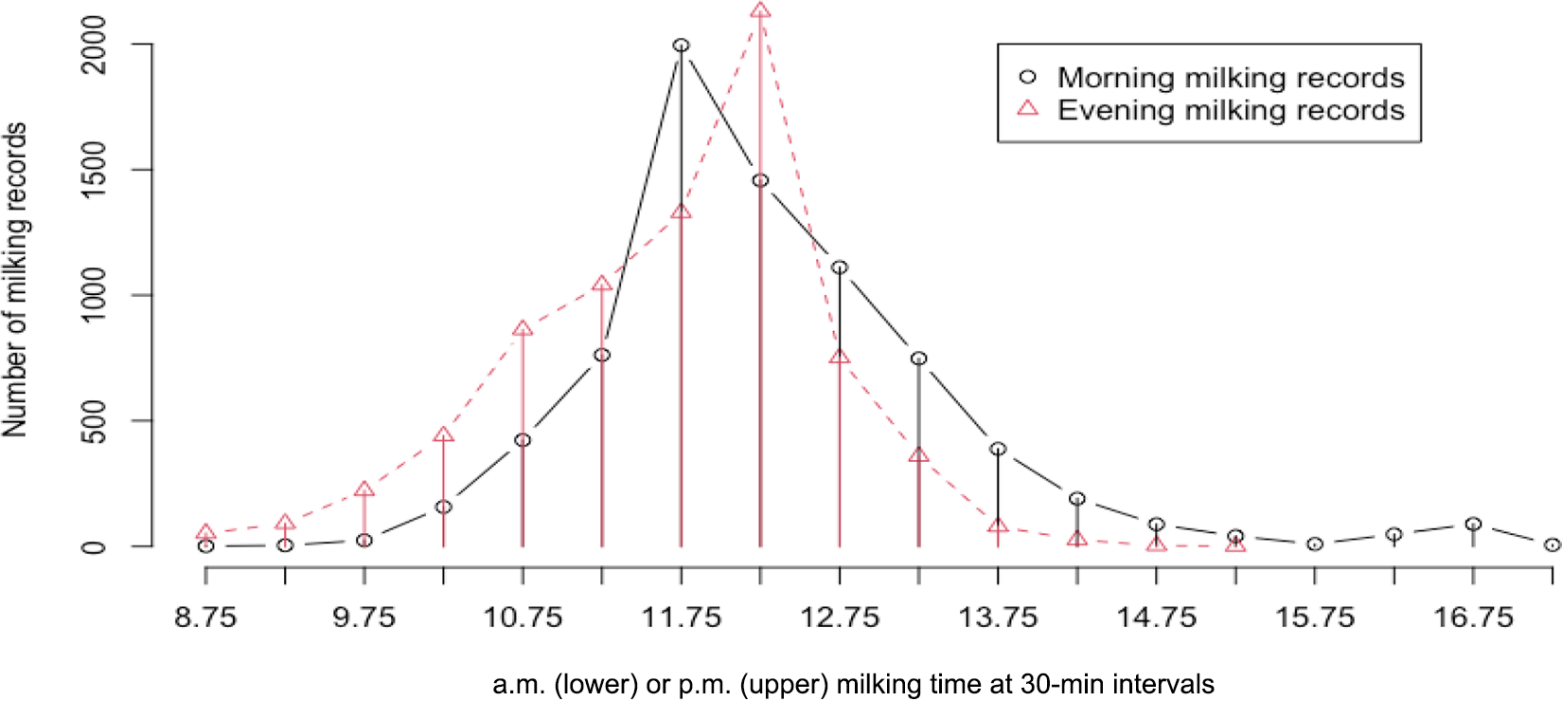
Distributions of milking records by months in milk and by milking interval time in 30-min intervals.

The polynomial-interaction regression models were implemented under 2 scenarios. The model in the first scenario (**PIR**) had milking interval time and partial (a.m. or p.m.) yields as the predictor variables. In the second scenario, the model included months in milk, parities, and geological regions (**MPR**) as additional predictor variables, thus denoted as **PIR_MPR**. Here, we approximated the categorical effect of months in milk, bypassing the complexity of the nonlinear effects of DIM (Wu et al., 2023b). For comparison, benchmark models included the D-W model, the George Wiggans (1986) model (denoted as **GW**), and linear regression (Liu et al., 2000). The D-W and GW models represented traditional MCF models without considering additional predictor variables. Similar to the PIR models, linear regression was implemented in 2 scenarios: one without MPR (denoted as **LR**) and the other with MPR (denoted as **LR_MPR**).

Model comparison was conducted using bootstrapping samples and validation samples. Each bootstrapping sample was generated by sampling with replacements, maintaining the same number of milking records as the entire dataset, based on unique cows. The unsampled milking records served as the validation set. Bootstrapping was randomly repeated 100 times, producing 100 bootstrapping samples and 100 validation samples. The performance of these models was evaluated and compared using 3 criteria: mean squared errors (**MSE**), correlation between actual and estimated daily milk yields, and R^2^ accuracy as defined below:

[8] $$R^{2}=1-\frac{\sum_{i=1}^{n}\;{\left(y_{i}-{\hat{y}}_{i}\right)}^{2}}{\sum_{i=1}^{n}\;{\left(y_{i}-\bar{y}\right)}^{2}},$$

where *y_i_* and ${\hat{y}}_{i}$ stand for the actual and estimated daily milk yields for the i-th animal, respectively, and $\bar{y}$ is the mean actual yield. Statistically, R^2^ measures the proportion of variance in the dependent variable that is predictable from the independent variable(s).

Discrete and continuous MCF were obtained and compared between the D-W and PIR models and compared (see the Graphical Abstract figure, middle). For the D-W model without the smoothing step, *F_k_* varied from 1.40 to 2.23 for a.m. MICL and from 1.73 to 3.37 for p.m. MICL. These *F_k_* were discrete and fluctuated substantially between neighboring MICL. Another challenge with the D-W model was insufficient data to compute MCF reliably for all MICL. We arbitrarily set the MCF as the mean of all MCF for a MICL with less than 20 milking records so that they stand identifiable as “outliers.” In practice, “missing MCF” are imputed by linear or quadratic smoothing instead (Shook et al., 1980; DeLorenzo and Wiggans, 1986). The D-W approach with follow-up smoothing also yielded discrete MCF because it computed MCF on the midpoints of each MICL. The MCF obtained from the 2 D-W models, implemented with or without the smoothing step, generally agreed with each other. However, there were noticeable discrepancies between both approaches for milking interval classes with insufficient data and for short (less than 9 h) or long (greater than 15 h) milking intervals. The latter differences were due to the quadratic smoothing with the D-W_1 model. In contrast, the PIR model produced a smooth curve of MCF as they were derived from a global model, simultaneously fitted all the data, and evaluated on every milking interval time unit.

Compared with the traditional D-W method, the renovated models (PIR and PIR_MPR) had smaller MSE and greater R^2^ accuracies (Table 1). The renovated models had an approximately 4.0% to 15.0% reduction in MSE and an up to around 2% increment in R^2^ accuracy. The increase in R^2^ accuracy was more significant with uneven a.m. and p.m. milking intervals and more pronounced at individual levels. For the latter comparison, we computed individual R^2^ accuracy. Compared with the D-W model, the PIR_MPR models had 0.24% of the animals showing ≥1% improvement in individual R^2^ accuracy and 0.06% showing ≥2% improvement in individual R^2^ accuracy. The maximum improvement in individual R^2^ accuracy was 4.10%. In contrast, the maximum negative change in individual R^2^ accuracy, favoring the D-W model, was −0.67%, meaning that no animals had an individual R^2^ accuracy from the D-W model that was 1% higher than that from the PIR_MPR model. The GW model performed roughly similarly to the D-W model. The LR model without MPR slightly outperformed the PIR model concerning MSE and R^2^. Nevertheless, the PIR model with MPR outperformed LR with MPR. In all the 6 models, PIR_MPR had the smallest MSE and the greatest R^2^. Mean squared errors decreased with lactation months, whereas correlations varied only slightly (Figure 2). The R^2^ accuracies were relatively higher in the beginning (1–3) and ending months (9–11+) of lactation because the daily yield variances were higher in these months compared with lactation mo 4 to 8.

**Table 1:** Comparing accuracies of estimated daily milk yields using 6 models^1^, ^2^

Model	Bootstrapping			Validation		
	MSE	R^2^	COR	MSE	R^2^	COR
D-W	10.7 (0.03)	0.897 (0.004)	0.950 (0.002)	11.0 (0.58)	0.895 (0.006)	0.950 (0.003)
GW	10.9 (0.03)	0.895 (0.004)	0.951 (0.002)	11.0 (0.56)	0.895 (0.006)	0.951 (0.002)
LR	10.2 (0.03)	0.902 (0.004)	0.949 (0.002)	10.2 (0.46)	0.902 (0.005)	0.950 (0.002)
LR_MPR	9.76 (0.03)	0.907 (0.003)	0.952 (0.002)	9.79 (0.42)	0.906 (0.004)	0.952 (0.002)
PIR	10.3 (0.03)	0.901 (0.004)	0.951 (0.002)	10.4 (0.55)	0.901 (0.006)	0.951 (0.002)
PIR_MPR	9.31 (0.03)	0.911 (0.003)	0.955 (0.002)	9.41 (0.42)	0.910 (0.004)	0.954 (0.002)

1 MSE = mean squared errors; COR = correlation between estimated and actual daily milk yields; $R^{2}=1-\frac{\sum_{i=1}^{n}\;{\left(y_{i}-{\hat{y}}_{i}\right)}^{2}}{\sum_{i=1}^{n}\;{\left(y_{i}-{\bar{y}}_{i}\right)}^{2}},$ where *y_i_* and ${\hat{y}}_{i}$ stand for the actual and estimated daily milk yields for the *i*th animal.

2 D-W = DeLorenzo-Wiggans (1986) model; GW = Wiggans (1986) model; LR = linear regression (Liu et al., 2000); LR_MPR = linear regression accounting for months in milk, parities, and geological regions (MPR); PIR = polynomial-interaction regression; PIR_MPR = PIR accounting for MPR.

**Figure 2:**
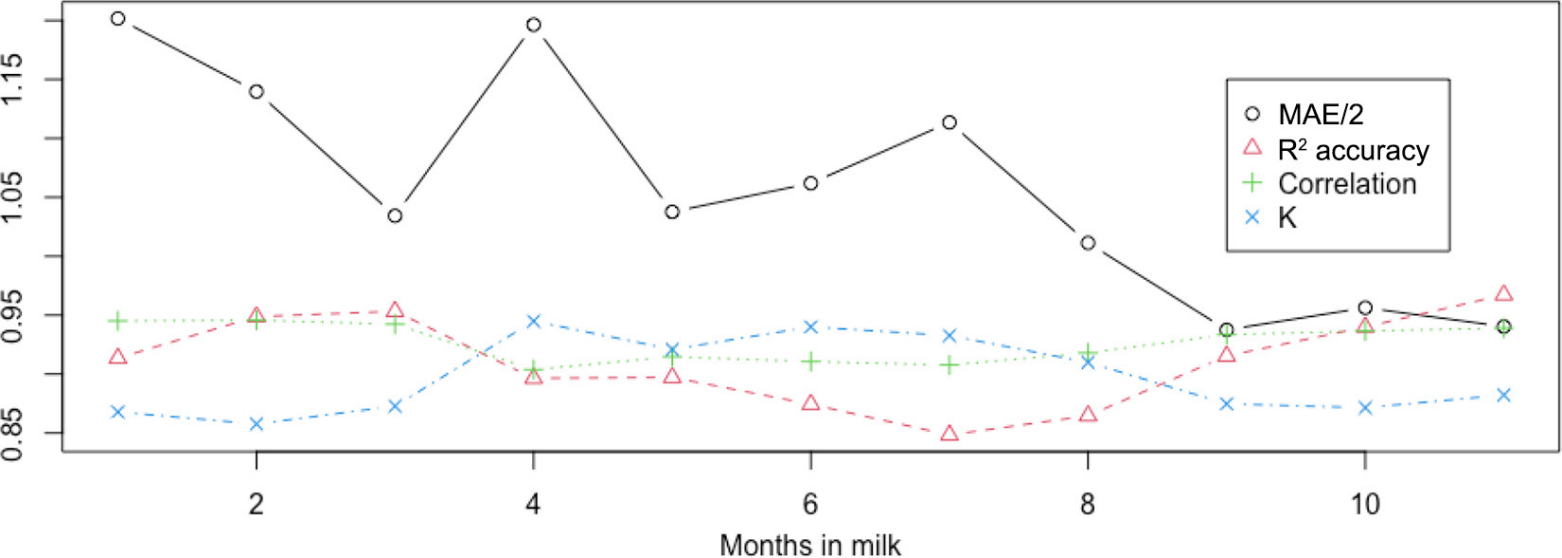
Comparing mean absolute errors (MAE/2) and R^2^ accuracies of estimated daily milk yields using 6 methods. Correlation = correlation between estimated and actual test-day milk yields; K = ratio of estimated to actual test-day milk yield variance.

Without accounting for MPR, LR had slightly greater R^2^ accuracy than the 2 traditional MCF models and the PIR model. Yet, it had the smallest correlations among the 6 models, leading to inconsistent conclusions. Statistically, correlation is a measure of associations between 2 quantities, not a precise measure of errors. For instance, let the correlation be $r=cor\left(y,\hat{y}\right).$ Then, adding a nonzero constant (Δ) to all the predicted values increases the errors, but it does not change the correlation: $cor\left(y,\hat{y}+\Delta \right)=cor\left(y,\hat{y}\right)=r.$

Discrete MCF introduces biases and, therefore, leads to more significant errors. Consider an animal, say *i*, with a partial yield collected for milking interval time *t*. Let the MCF be a quadratic polynomial function of milking interval time. A precise estimate of MCF for milking interval time *t* is the following:

[9] $$\begin{array}{l} F_{t}=b_{0}+b_{1}t+b_{2}t^{2}\\ =b_{0}+b_{1}\left[\bar{t}+\left(t-\bar{t}\right)\right]+b_{2}{\left[\bar{t}+\left(t-\bar{t}\right)\right]}^{2}\\ =\left(b_{0}+b_{1}\bar{t}+b_{2}{\bar{t}}^{2}\right)+\left[b_{1}\left(t-\bar{t}\right)-2b_{2}\left(t-\bar{t}\right)+b_{2}{\left(t-\bar{t}\right)}^{2}\right]. \end{array}$$

In the above, $t\neq \bar{t},$ and $\bar{t}$ is the midpoint time in that milking interval class. Then, the daily milk yield is estimated by

[10] $${\hat{y}}_{ik}=F_{k}x_{ik}+\left[{\hat{b}}_{1}\left(t-\bar{t}\right)-2{\hat{b}}_{2}\left(t-\bar{t}\right)+{\hat{b}}_{2}{\left(t-\bar{t}\right)}^{2}\right]x_{ik},$$

where $F_{k}={\hat{b}}_{0}+{\hat{b}}_{1}\bar{t}+{\hat{b}}_{2}{\bar{t}}^{2}.$ The traditional MCF model estimated the daily milk yield as ${\hat{y}}_{ik}\approx F_{k}x_{ik},$ which corresponds to the first term on the right-hand side of the equation [10]. Yet, it ignores the second term on the right-hand side, which then becomes the bias.

The bottom left figure in the Graphical Abstract shows the R^2^ accuracies obtained from the D-W model with varying MICL size. The R^2^ accuracy decreased as the MICL size increased from 0.05 to 1 h. When comparing the test-day yield estimates obtained from the discrete MCF model (D-W) and the continuous MCF model (PIR), the discrepancies in yield estimates between the 2 models showed recurrent patterns. The difference was minimum at the midpoint of each MICL, increased as the milking interval moved off the midpoint, and reached the maximum at the boundaries between 2 MCF.

The estimation accuracy was also evaluated by regressing the actual daily milk yields on the estimated daily milk yields for 3 models, D-W, PIR, and PIR_MPR, which were assessed on the entire milking dataset. The PIR model gave more accurate yield estimates than the D-W model. The intercept (*â*) represents a systematic baseline bias, which was higher (2.60 ∼3.28) for the D-W model than for the PIR model (2.56 ∼3.24). The larger intercept resulted from omitting the intercept in the prediction model. The regression coefficients $\left(\hat{b}\right)$ were all smaller than 1, indicating that both models overestimated daily milk yields compared with the actual yields. The D-W model overestimated daily milk yields more than the PIR model because it had smaller regression coefficients (0.906 ∼0.925) than the PIR model (0.907 ∼0.927). For $y=a+b\hat{y}+e,$ we have $Var\left(y\right)={\hat{b}}^{2}Var\left(\hat{y}\right)+Var\left(e\right).$ To satisfy this relation, the regression coefficient ought to be smaller than 1 if $Var\left(\hat{y}\right)>Var\left(y\right).$ The actual variance was 104.2. In contrast, the variance of estimated daily yields was 110.2 (a.m.) to 114.2 (p.m.) for the D-W model and 109.9 (a.m.) to 114.2 (p.m.) for the PIR model. For the PIR_MPR model accounting for additional covariates and categorical regressor factors, the intercepts were close to 0, and the regression coefficients were close to 1. The PIR_MPR model does not include an intercept either. However, the categorical MPR effects in the PIR_MPR models were estimated by the effect of each category directly on the dependent variable, inherently compounded with the omitted intercept. The approximately 0 intercept and unity regression coefficients suggested that the PIR_MPR model empirically provided unbiased estimates of daily milk yields in a way that captured the central tendency of actual daily yields. However, it did not account for all the variability because estimated yields had a smaller variance (94.8–94.6) than the actual variance. For the PIR_MPR model, because $\hat{b}=1,$ we observe $Var\left(y\right)=Var\left(\hat{y}\right)+Var\left(e\right),$ where $Var\left(\hat{y}\right)<Var\left(y\right)$ and *Var*(*e*) > 0. Similarly, linear regression with intercept also gave estimates with a smaller variance than actual yields.

In the present study, accommodating more covariates and factors with LR and PIR significantly decreased MSE and improved R^2^ accuracies. This was evidence that the data blending was not “perfect.” The PIR_MPR outperforms LR_MPR, possibly because the former captured the interactions between partial yields and milk interval time. Analysis of variance based on the reparametrized polynomial-interaction model with MPR effects showed a highly significant impact of partial yields on test-day yields (*P* <2.2e-16). The interaction effect between a partial yield and linear milking interval time was highly significant (*P* < 2.2e-16). The interaction effect between a partial yield and quadratic milking interval time was suggestive (*P* = 0.081) for a.m. milkings and highly significant (*P* <2.2e-16) for p.m. milkings. These findings support the inclusion of interactions in the modified D-W model. Months in milk significantly affected daily milk yields (*P* < 2.2e-16). The ANOVA results also showed highly significant parity effects (a.m.: *P* = 1.76e-07; p.m.: *P* = 2.18e-04) and regional effects (a.m.: *P* = 1.44e-07; p.m. <2.2e-16) on daily milk yields. In the present study, the MPR variables are considered secondary. Accounting for the difference due to the secondary predictor variables can improve the accuracy when the blending is unsuccessful.

Finally, the PIR model has no intercept. This model setting is subject to the criticism that it would unrealistically enforce *x* = 0 and *y* = 0 (Liu et al., 2000). Statistically, linear regression with an intercept would allow the model to fit the data flexibly. In particular, linear regression with an intercept can be more useful when the dependent variable does not naturally pass through the origin when *x* = 0. It can also account for any baseline level of *y* independent of *x*. Probably because of the above reasons, the PIR without intercept performed slightly worse than the LR with intercept in terms of MSE and R^2^ accuracies. Also, it should be noted that the R^2^ accuracy defined in [8] is appropriate for linear regression with an intercept in the sense that the variance of estimates is smaller than the total (actual) variance. A more appropriate accuracy measure for linear regression with an intercept would be the R^2^, which resembles the liability measure (Wu et al., 2023b) because the estimated variance is often greater than the actual variance. It should be noted that the latter measure could give slightly greater accuracy for the MCF models.

In theory, the purpose of omitting the intercept is to produce MCF instead of ACF (Wu et al., 2023a). Its primary impact was observed in the variance of estimated daily milk yields. A linear model with an intercept tends to generate a smaller estimate variance than the actual variance. Again, let *y* = *a* + *bx* + *e*. We have $Var\left(\hat{y}\right)$ = ${\hat{b}}^{2}Var\left(x\right)$ ≤ Var(y) = $b^{2}Var\left(x\right)+Var\left(e\right),$assuming $\hat{b}=b.$ In contrast, the model without intercept can inflate the variance of estimates due to reasons such as lack of centering of predictor variables, multicollinearity, inappropriate scaling and nonzero means of predictor variables, and heteroscedasticity of the residuals. Often, omitting the intercept leads to a significantly larger $\hat{b},$ which, in turn, can give a larger estimate variance than the actual variance. When we evaluated the variance ratio per month in milk across the entire dataset, the mean (range) of the variance ratios were 1.134 (1.086–1.183) with D-W, 1.157 (1.103–1.215) with GW, 0.950 (0.909–0.998) with LR, 1.132 (1.076–1.192) with PIR, 0.895 (0.856–0.944) with LR_MPR, and 0.898 (0.858–0.945) with PIR_MPR. In practice, either reduced or inflated variance of estimated daily milk yields has a nonignorable impact on genetic evaluations because it can lead to varied genetic variance. Therefore, estimated daily milk yields must be rescaled to be comparable to the actual variance. A simple practice is to apply a common rescaling factor equaling the square root of the estimated to actual daily yield variance ratio. Down-scaling the variance often reduces the errors, whereas inflating the variance tends to increase the errors further. In the study, rescaling the variance reversed the conclusion of the model comparison: PIR had around 1% higher R^2^ accuracy than LR.

In conclusion, we proposed a strategy to incorporate continuous MCF into the established methods, exemplified by the DeLorenzo-Wiggans (1986) model. The traditional D-W method represents a 2-step procedure, where the smoothing uses data information captured only by means, possibly leading to oversimplified and potentially biased results due to the loss of detailed information. In contrast, the renovated D-W method fully leveraged the available data and better captured the underlying relationship between the variables through modeling interactions between partial yields and milking interval time. The latter formed the basis for accurately computing MCF and estimating daily milk yields. Utilizing all available data information also allows for more accurate estimating of the error term, which is crucial for understanding the model's predictive accuracy and any statistical tests. We demonstrated the methodology with the a.m.-p.m. milking plans, yet this modeling strategy is universally applicable across various milking plans, subject to some necessary modifications.
